# Estimating Morbidity Rates Based on Routine Electronic Health Records in Primary Care: Observational Study

**DOI:** 10.2196/11929

**Published:** 2019-07-26

**Authors:** Mark M J Nielen, Inge Spronk, Rodrigo Davids, Joke C Korevaar, René Poos, Nancy Hoeymans, Wim Opstelten, Marianne A B van der Sande, Marion C J Biermans, Francois G Schellevis, Robert A Verheij

**Affiliations:** 1 Netherlands Institute for Health Services Research Utrecht Netherlands; 2 Centre for Health and Society National Institute for Public Health and the Environment Bilthoven Netherlands; 3 Dutch College of General Practitioners Utrecht Netherlands; 4 Centre for Infectious Disease Control National Institute for Public Health and the Environment Bilthoven Netherlands; 5 Julius Center for Health Sciences and Primary Care Julius Global Health Utrecht Netherlands; 6 Department of Primary and Community Care Radboud University Medical Center Nijmegen Netherlands

**Keywords:** morbidity, primary care, electronic health records, episode of illness

## Abstract

**Background:**

Routinely recorded electronic health records (EHRs) from general practitioners (GPs) are increasingly available and provide valuable data for estimating incidence and prevalence rates of diseases in the population. This paper describes how we developed an algorithm to construct episodes of illness based on EHR data to calculate morbidity rates.

**Objective:**

The goal of the research was to develop a simple and uniform algorithm to construct episodes of illness based on electronic health record data and develop a method to calculate morbidity rates based on these episodes of illness.

**Methods:**

The algorithm was developed in discussion rounds with two expert groups and tested with data from the Netherlands Institute for Health Services Research Primary Care Database, which consisted of a representative sample of 219 general practices covering a total population of 867,140 listed patients in 2012.

**Results:**

All 685 symptoms and diseases in the International Classification of Primary Care version 1 were categorized as acute symptoms and diseases, long-lasting reversible diseases, or chronic diseases. For the nonchronic diseases, a contact-free interval (the period in which it is likely that a patient will visit the GP again if a medical complaint persists) was defined. The constructed episode of illness starts with the date of diagnosis and ends at the time of the last encounter plus half of the duration of the contact-free interval. Chronic diseases were considered irreversible and for these diseases no contact-free interval was needed.

**Conclusions:**

An algorithm was developed to construct episodes of illness based on routinely recorded EHR data to estimate morbidity rates. The algorithm constitutes a simple and uniform way of using EHR data and can easily be applied in other registries.

## Introduction

Data from electronic health records (EHRs) are increasingly used for clinical and epidemiological research. Compared with the more traditional research designs such as clinical trials and cohort studies, the use of EHRs as a data source for research has major advantages, including using large study populations for lower costs and decreasing timelines of studies. However, researchers must overcome problems regarding completeness of data, data quality, and absence of data for patients who do not visit their health professional on a regular basis. To deal with imperfect data from EHRs, algorithms are needed to make EHR data useful for clinical research. In this study, we developed a method to estimate countrywide morbidity rates based on EHRs of general practitioners (GPs). Morbidity estimates are a key element in the establishment of a learning health care system [1-3]. Valid estimations of morbidity rates in the general population are essential for patient management by health care providers, developing and evaluating health care policy, and providing input for research. Many European countries, including the Netherlands and the United Kingdom, already have a long history of using EHRs of GPs as a data source for morbidity estimates [1,2,4-6].

The extent to which EHRs of GPs are a valid data source to assess the health status of the general population depends on how primary care is organized in a country. Important primary care characteristics for calculating valid morbidity rates include (1) free access to primary care, (2) first presentation of health problems in general practice, (3) uniform coding system for recording diagnoses and symptoms, and (4) valid information about epidemiological denominator based on a fixed patient list or method to estimate the patient list [7]. Dutch primary care meets all these requirements, since, like in many other European countries, the GP has a gatekeeper role for specialized care and is the first professional to be consulted for health problems. According to the Dutch College of General Practitioners, all GPs are expected to routinely record diagnostic information from their patients using the International Classification of Primary Care version 1 (ICPC-1) [8]. All noninstitutionalized Dutch inhabitants are compulsorily listed with a general practice, including patients who do not visit their GP on a regular basis. Based on these primary care characteristics, data from Dutch EHRs are a good foundation for developing a methodology for population-based estimations of morbidity.

In 2009, the Dutch College of General Practitioners published a guideline about adequate recording of medical information in EHRs to promote uniform, complete, and good quality recording in general practice [9]. This guideline and the development of a feedback tool for GPs with information about the quality of data in their EHRs [10], among other things, resulted in improved quality of diagnosis recording. According to the guideline, GPs should structure their EHRs around episodes of care that contain all patient encounters, prescribed medication, and interventions related to the same health problem. As a result, all relevant information is structured together by disease, which also makes it easier to exchange information between health care providers.

Episodes of care could form the basis of calculating morbidity rates. However, several steps are needed to convert episodes of care from EHRs into morbidity rates. First, the last contact in an episode of care is, in general, not the moment when the patient is considered to be cured. Patients only consult their GP when they experience a health problem and seldom inform the doctor when they are cured. A valid estimation of the start and stop date of an episode is essential to determine whether an episode is new or existing in a certain period to establish a numerator for morbidity rates and determine whether a patient is at risk for a specific disease (necessary to assess the denominator for incidence rates). Therefore, instead of episodes of care, episodes of illness, which “extend from the onset of symptoms to their complete resolution” [11], are required for valid morbidity rates. To construct episodes of illness from recorded episodes of care, a stop date of the episode of illness should be estimated based on knowledge of the duration of a disease. Second, there are problems related to recording habits of GPs that need to be solved in the process of constructing episodes of illness, since GPs do not always record clinical items adequately in the EHR [12]. There is a wide variety in the way the concept of episodes is implemented in the recording habits of GPs because the rationale behind recording diagnoses in EHRs is not to facilitate research but facilitate patient care. For example, many GPs collapse multiple episodes of illness into one episode of care in their EHR systems. Also, not all encounters are recorded within an episode of care, since GPs can choose to record encounters separately, and it is questionable whether all encounters are recorded within the correct episode of care. Finally, after constructing episodes of illness, the numerator and denominator need to be defined to calculate incidence and prevalence rates. A previous study showed a large amount of variation in morbidity estimates between different registries [13]. One of the reasons for these variations may be different ways of calculating morbidity rates. Such differences may also result in unexplainable international variations [4].

The aim of this study was to (1) develop a simple and uniform algorithm to construct episodes of illness based on EHR data, (2) develop a method to calculate morbidity rates based on these episodes of illness, and (3) discuss how this algorithm can be used in other settings. In addition, we determined the influence of using constructed episodes of illness instead of recorded episodes of care.

## Methods

### Development of the Algorithm to Construct Episodes of Illness

The algorithm to construct episodes of illness, based on EHRs of GPs, was developed by two expert groups. The first expert group, consisting of two GPs and five epidemiologists from the Netherlands Institute for Health Services Research (NIVEL), made the draft of the algorithm. Decisions were made about how to (1) estimate the stop date of the episode of illness for all symptoms and diseases in ICPC-1 [8], (2) construct episodes of illness based on encounters not recorded in episodes of care, and (3) deal with encounters recorded in a nonappropriate episode of care. The algorithm was finalized by a second group of experts with researchers; epidemiologists; GPs; and medical informaticians from NIVEL, National Institute for Public Health and the Environment (RIVM), Dutch College of General Practitioners, and Radboud University Medical Center. During this meeting, all previous steps were evaluated, the algorithm was finalized, and a method was developed for calculating incidence and prevalence rates based on the constructed episodes of illness.

### Study Setting: Netherlands Institute for Health Services Research Primary Care Database

The algorithm was developed in a dataset from the NIVEL Primary Care Database (NIVEL-PCD), including a representative sample of 219 general practices covering a total population of 867,140 listed patients [14]. NIVEL-PCD collects data from routine EHR systems including consultations, morbidity, prescriptions, and diagnostic tests. Diagnoses are recorded using the ICPC-1 coding system (Multimedia Appendix 1) [8]. All general practices in the sample had sufficient data quality over the period 2010-2012, fulfilling the following criteria: at least 500 listed patients, complete morbidity registration (defined as 46 or more weeks per year; this is, a year minus a maximum of six weeks’ holidays), and sufficient ICPC coding of diagnostic information (defined as 70% or more of recorded encounters with an ICPC code) [15]. Morbidity data used included ICPC-coded episodes of care, encounters, and diagnosis-coded prescriptions.

Dutch law allows the use of extractions of EHRs for research purposes under certain conditions. According to Dutch legislation, obtaining neither informed consent nor approval by a medical ethics committee is obligatory for this kind of observational study [16].

### Statistical Analyses

Episodes of illness were constructed according to the algorithm within the NIVEL-PCD using structured query language. Incidence and prevalence rates were calculated with Stata 13 software (StataCorp LLC). The influence of the algorithm on morbidity rates was tested in two analyses. First, for the most common symptoms and diseases we compared the number and average duration of the recorded episodes of care and constructed episodes of illness in 2012. Only episodes of illness with a stop date after December 31, 2011, were selected. For calculating the average episode duration, only the number of days of an episode in the year 2012 was used. Second, we tested the influence of using different estimates of the stop date of the episodes of illness on incidence and prevalence rates.

## Results

### Algorithm to Construct Episodes of Illness

**Figure 1:**
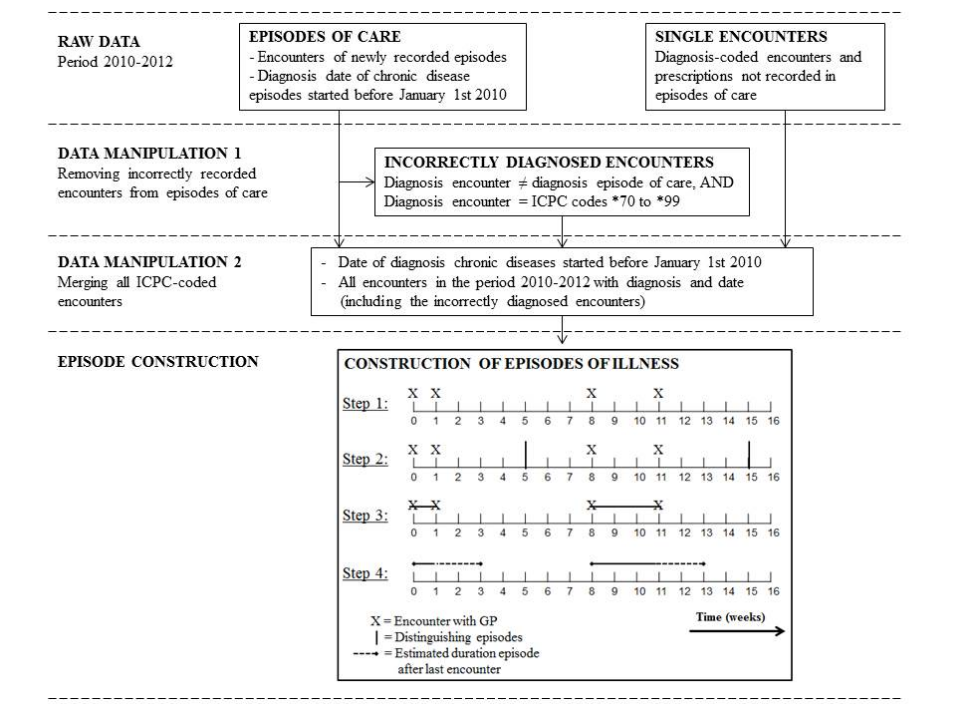
Algorithm to construct episodes of illness based on recorded data from electronic health records. GP: general practitioner; ICPC: International Classification of Primary Care.

The developed algorithm, used to construct episodes of illness over the year 2012, is shown in Figure 1. The input for the algorithm consisted of raw data from EHRs over the period 2010-2012, including encounters recorded in episodes of care, single diagnosis-coded encounters, and dates of diagnosis for all chronic diseases that started before January 1, 2010. Recorded start dates of an episode of care were regarded as an encounter, even if there was no patient contact recorded on that day. This may, for instance, be the case when the GP records an episode of care based on information from another health professional, such as a letter from a medical specialist without the patient consulting the GP. We have chosen to include data before the year 2012 for a correct estimate of episodes of illness that started in previous years. The raw recorded data resulted in constructed episodes of illness following a number of steps.

Incorrectly recorded encounters were removed from the episodes of care (step 1). It is possible to record an encounter with a certain ICPC code within an episode of care having another ICPC code (eg, recording an encounter for coughing, ICPC R05, in the episode of care asthma, ICPC R96). ICPC-1 consists of symptom codes (ICPC codes *01 to *29) and disease codes (ICPC codes *70 to *99). Since it seems correct to record symptoms of a disease within an episode of care of this particular disease, disease codes were only regarded as incorrectly recorded if reported within another episode of care for a disease. This was the case in 280,657 coded encounters and prescriptions in the period 2010-2012, 4.4% of the total number of ICPC-coded encounters and prescriptions. Encounters most commonly recorded incorrectly were for hypertension, acute upper respiratory infection, diabetes mellitus, cystitis/other urinary infection, and contact dermatitis/allergic eczema.

All diagnosis-coded encounters and prescriptions were merged into one data file (step 2). This data file contained all ICPC-coded encounters and prescriptions in the period 2010-2012. For all correctly recorded encounters (and prescriptions) within the episode of care, the ICPC code of the episode of care was used. Hereafter, we added the encounters that were recorded in the incorrect episodes of care (see previous step) and all 736,381 single diagnosis-coded encounters and prescriptions, 4.7% of the total number of encounters in the period 2010-2012. Finally, all dates of diagnosis from chronic diseases that had started before January 1, 2010, were added.

Construction of episodes of illness was based on dates of encounters and prescriptions (step 3). For the construction of episodes of illness, the expert groups introduced the term *contact-free interval,* defined as “the period in which it is likely a patient will visit the GP again if a medical complaint persists.” After this interval, it is more likely that an encounter for this complaint constitutes a new episode of illness. The contact-free interval was based on expert opinion about the natural history of a disease and used to estimate the stop date of the episode of illness. During the expert meetings, all symptoms and diseases of ICPC-1 were categorized (Multimedia Appendix 2) in five disease groups with accompanying contact-free intervals: acute symptoms and diseases with short (4 weeks), moderate (8 weeks), and long (16 weeks) contact-free intervals; long-lasting reversible diseases (with 1-year contact-free intervals); and chronic diseases. Chronic diseases are considered irreversible, and no contact-free interval is needed. The list of 109 chronic diseases was based on both national and international literature [17,18]; the remaining ICPC codes were assigned to the other four categories in several discussion rounds. Chronic diseases included disabilities, congenital anomalies, malignant cancer, diabetes mellitus, hypertension, inflammatory arthritis, psoriatic disease, and dementia. Long-lasting reversible diseases included allergies, acute myocardial infarction, migraine, depression, and carpal tunnel syndrome. Acute symptoms and diseases included fever, vomiting, diarrhea, excessive ear wax, acute upper respiratory infection, insect bites, and laceration/cut.

Since chronic diseases are considered irreversible, chronic episodes of illness terminate only when a patient dies. For the construction of these episodes, only the start date of the episode is of interest, defined as the “start date of an episode of care or the first consultation or prescription for that specific disease.” An example of how the construction of episodes of illness works for acute symptoms and diseases and long-lasting reversible diseases is shown in Figure 1. In this example, the construction of episodes of illness is described for an acute disease with a contact-free interval of 4 weeks. A patient visits his GP for the complaint, followed with an encounter for the same complaint 1, 8, and 11 weeks later (step 1). The contact-free interval of 4 weeks means that a period of 4 weeks between two encounters results in the construction of a new episode. Applying this rule (step 2) results in closing an episode at weeks 5 and 15, respectively. This will result in two constructed episodes: episode 1 with the first encounter at t=0 and the last encounter at t=1 week and episode 2 with the first encounter at t=8 weeks and the last encounter at t=11 weeks (step 3). After the last encounter within an episode of illness, it is unclear how long it takes until the patient recovers. Since this will be between the time of the last encounter and the duration of the contact-free interval, half of the duration of the contact-free interval is added to the last encounter. In this example, this period is 2 weeks, resulting in constructed episodes of illness between weeks 0 and 3 and between weeks 8 and 13 (step 4).

### Method for Calculating Incidence and Prevalence Rates

**Figure 2:**
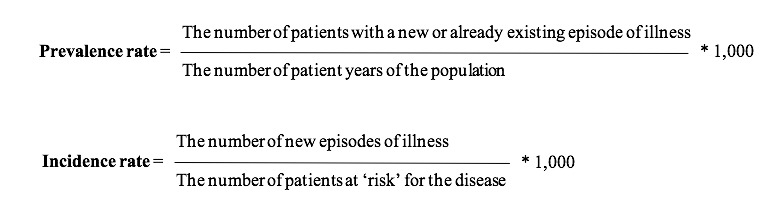
Prevalence and incidence rate equations.

After constructing episodes of illness over the period 2010-2012, all ongoing episodes and newly constructed episodes in 2012 can be used for calculating prevalence and incidence rates for 2012 (see Figure 2 for formulas).

Based on claims data from the EHR, we could determine for each quarter of a year whether an individual was part of the practice population. When there was no claim, we assumed the patient was no longer registered in the practice (eg, due to death or moving to another area). In 2012, the population consisted of 757,751 person years in the selected 219 general practices. For the denominator of the incidence rate we used the number of patients at risk for a particular disease, which is the number of person years of the total population minus the sum of the duration of all episodes in 2012. This definition was used for all long-lasting reversible diseases and chronic diseases. For all acute symptoms and diseases, we used the number of patient years in the studied population as denominator. Disease duration was not taken into account for acute symptoms and diseases since (1) the period at risk is almost equal to the total number of patient years due to the short episode duration and (2) patients are still at risk for the disease during an active episode in some cases, which is, for example, the case for bone fractures. Incidence and prevalence rates of all ICPC-coded symptoms and diseases are shown in Multimedia Appendix 2.

### Differences Between Recorded Episodes of Care and Constructed Episodes of Illness

The number and average duration of the recorded episodes of care and constructed episodes of illness for the five most common diseases per disease category in 2012 are shown in Table 1. For acute and long-lasting diseases, applying the algorithm resulted in a reduction of both the number and average duration of the episodes compared to the (recorded) episodes of care. In the three categories of acute symptoms and diseases, number of episodes decreased between 8.8% and 52.5% with a decrease of episode duration between 59.9% and 93.8%. For long-lasting diseases, reduction of the number of episodes was between 17.5% and 33.6% with a decrease of episode duration between 24.3% and 39.6%.

There are two main reasons for these reductions. First, in most cases an episode of care is not closed by a GP when a patient is cured but remains open or is automatically closed by the EHR system of the GP after a large amount of time. This results in a large number of episodes that started in previous years with an episode duration of the complete period of follow-up of the patient in 2012. Second, GPs can start several episodes of care for the same disease in the same time period, which is not possible with the algorithm.

On the other hand, for chronic diseases, the algorithm resulted in an increase in the number of episodes as well as episode durations. This was mainly caused by the construction of episodes of illness based on single encounters and incorrectly diagnosed encounters from episodes of care (Figure 1).

**Table 1:** Number and average duration of recorded episodes of care and constructed episodes of illness in 2012 (n=219 practices, n=757,751 person years).

Disease category			Constructed episodes of illness		Recorded episodes of care	
			Episodes, n	Episode duration (days), mean	Episodes, n	Episode duration (days), mean
**Acute symptoms/diseases (contact-free interval: 4 weeks) and ICPC^a^ code**						
	R74	Upper respiratory infection acute	70,914	17.3	103,433	253.7
	H81	Excessive ear wax	42,028	16.5	65,300	264.0
	L81	Other injury musculoskeletal system	21,553	18.2	34,917	240.5
	R21	Throat symptoms/complaints	18,421	17.0	35,806	262.0
	S18	Laceration/cut	17,344	18.1	36,501	253.0
**Acute symptoms/diseases (contact-free interval: 8 weeks) and ICPC code**						
	U71	Cystitis/other urinary infection	64,113	40.4	84,159	262.5
	R05	Cough	58,129	34.2	84,003	263.2
	S74	Dermatophytosis	40,971	34.6	66,606	262.4
	L03	Low back symptoms/complaints without radiation	37,567	38.1	59,445	270.6
	A04	General weakness/tiredness/ill-feeling (excluding psychological)	34,199	37.9	54,802	257.5
**Acute symptoms/diseases (contact-free interval: 16 weeks) and ICPC code**						
	D02	Stomach ache/stomach pain	27,125	111.2	29,738	282.7
	P06	Disturbances of sleep/insomnia	26,044	111.4	31,777	277.6
	P01	Feeling anxious/nervous/tense/inadequate	15,837	104.1	21,481	278.1
	S79	Other benign neoplasms of skin	15,369	59.5	28,443	255.7
	R81	Pneumonia	13,915	66.0	19,969	252.9
**Long-lasting reversible diseases (contact-free interval: 1 year) and ICPC code**						
	S88	Contact dermatitis/allergic eczema	48,961	169.5	67,767	274.5
	L99	Other disease musculoskeletal system/connective tissue	44,289	158.5	65,279	262.2
	R97	Hayfever/allergic rhinitis	40,200	223.4	60,557	296.0
	W11	Family planning/oral contraception	36,689	210.8	45,260	278.4
	D12	Constipation	31,482	179.3	38,183	258.0
**Chronic diseases and ICPC code**						
	K86	Uncomplicated hypertension	116,173	339.1	102,786	310.3
	R96	Asthma	73,018	334.9	57,188	302.1
	S87	Atopic dermatitis/other eczema	69,023	328.5	40,425	278.5
	T93	Lipid metabolism disorder	58,755	334.2	44,363	301.2
	T90	Diabetes mellitus	52,174	337.2	52,949	313.8

^a^ICPC: International Classification of Primary Care.

### Influence of the Contact-Free Interval on Incidence and Prevalence Rates

For the most common acute symptoms and diseases, incidence and prevalence rates were calculated using contact-free intervals of 4 weeks, 8 weeks, and 16 weeks (Table 2). In general, increasing the contact-free interval resulted in a decrease of the incidence, which is caused by reducing the number of constructed episodes of illness. The longer the contact-free interval, the higher the chance that encounters are combined in one episode rather than several episodes. On the other hand, the prevalence rate increased when the contact-free interval increased. When the length of an episode increases, the number of disease episodes that started in a previous year are used in the calculation of the prevalence rate, resulting in higher rates.

**Table 2:** Incidence and prevalence rates of acute symptoms and diseases when using different contact-free intervals.

Disease category				Incidence rate (per 1000 person years)					Prevalence rate (per 1000 person years)				
				4 weeks		8 weeks		16 weeks		4 weeks		8 weeks	16 weeks
**Acute symptoms/diseases (contact-free interval: 4 weeks) and ICPC^a^ code**													
	R74	Upper respiratory infection acute	60.6		55.7		50.3		54.9		57.3		61.6
	H81	Excessive ear wax	37.5		36.3		34.5		37.3		38.4		40.5
	L81	Other injury musculoskeletal system	17.9		16.6		15.4		17.4		18.0		19.4
	R21	Throat symptoms/complaints	18.0		16.7		15.4		16.6		17.2		18.5
	S18	Laceration/cut	15.7		15.1		14.6		16.9		17.4		18.5
**Acute symptoms/diseases (contact-free interval: 8 weeks) and ICPC code**													
	U71	Cystitis/other urinary infection	60.7		52.9		44.7		49.1		50.5		53.4
	R05	Cough	54.0		48.5		42.9		48.4		50.5		54.3
	S74	Dermatophytosis	38.9		35.1		31.3		33.5		34.6		36.9
	L03	Low back symptoms/complaints without radiation	39.2		33.7		28.7		30.4		31.5		33.8
	A04	General weakness/tiredness/ill-feeling (excluding psychological)	32.7		29.1		25.8		29.6		30.5		32.5
**Acute symptoms/diseases (contact-free interval: 16 weeks) and ICPC code**													
	D02	Stomach ache/stomach pain	41.0		34.6		19.1		21.8		22.4		23.5
	P06	Disturbances of sleep/insomnia	42.9		27.6		18.6		21.7		22.3		23.3
	P01	Feeling anxious/nervous/tense/inadequate	24.8		17.3		12.1		13.7		14.0		14.7
	S79	Other benign neoplasms of skin	13.4		12.5		11.8		13.1		13.4		14.4
	R81	Pneumonia	11.5		10.1		9.0		10.7		11.2		12.1

^a^ICPC: International Classification of Primary Care.

For long-lasting reversible diseases, incidence and prevalence rates were calculated with a contact-free interval of 1 and 2 years, respectively. Since there were almost no differences in incidence rates between the two contact-free intervals, we chose a contact-free interval of 1 year for all long-lasting reversible diseases (data not shown). Compared with a period of 2 years, the chance of overestimating the episode length is much smaller with a contact-free interval of 1 year and the half year (half of the duration of the contact-free interval) that is added to the last encounter.

## Discussion

### Principal Findings

In this study, we developed an algorithm to construct episodes of illness based on routinely recorded EHR data to estimate morbidity rates. All 685 symptoms and diseases of ICPC-1 were categorized as acute symptoms and diseases, long-lasting reversible diseases, or chronic diseases. Compared with recorded episodes of care, applying the algorithm for acute and long-lasting diseases resulted in a reduction of the number and average duration of episodes up to 53% and 94%, respectively. On the other hand, for chronic diseases, the algorithm resulted in a slight increase in the number of episodes and episode durations.

The potential of using routine EHR data for epidemiology and health policy is enormous. Routine health data are regarded as a means to arrive at a rapid learning health care system, a system “in which knowledge generation is so embedded into the core of the practice of medicine that it is a natural outgrowth and product of the health care delivery process and leads to continual improvement in care” [19]. However, to use this potential we need sound methodologies to turn these huge amounts of raw data into meaningful information. In this study, we developed a simple and uniform algorithm to construct episodes of illness based on routine primary care EHR data, making it possible to estimate incidence and prevalence rates of symptoms and diseases. Compared with other methods such as questionnaires and cohort studies, the use of EHRs from GPs has a number of advantages: (1) diagnoses are made by a health professional, (2) GPs have an excellent overview of all morbidity presented to them in their patient population, (3) because of the fixed patient list, there is also information available on healthy individuals who do not visit their GP on a regular basis, (4) the populations listed in general practices are representative of the general population, and (5) due to the large number of patients, it is possible to give reliable estimates of low prevalence diseases.

### Comparison With Prior Work

Verheij et al [20] recently described a number of factors that can influence the results of studies based on EHRs, including the way health care professionals record information in EHRs, differences between EHR systems, methods used to extract information from EHR systems, and how the data are used by a data analyst and researcher. All these factors together make it difficult to make a fair comparison between our morbidity rates and rates from other Dutch studies. However, the developed algorithm to construct episodes of illness can be compared with the method we used previously in NIVEL-PCD: the Episode Constructor (EPICON) method [6,21,22]. Before 2012, NIVEL-PCD used the EPICON method to group recorded diagnoses into episodes of care for estimating morbidity rates. However, grouping diagnoses into episodes of care is no longer needed, since GPs are already recording episodes of care in their EHRs [9]. Furthermore, converting episodes of care into episodes of illness with the algorithm results in a more valid estimation of morbidity rates. An episode of care is “the period from the first presentation of a health problem or illness to a health care provider until the completion of the last encounter,” whereas episodes of illness “extend from the onset of symptoms to their complete resolution” [11]. Based on these definitions, it was expected that the episode of care has a shorter duration compared with the episode of illness, since in general a disease is not cured at the last encounter. However, applying the algorithm resulted in a reduction of the average episode duration. In most cases, a recorded episode of care was not closed by a GP when the patient was cured but remained open or was automatically closed by the EHR system of the GP after a large amount of time. Also, instead of constructing episodes of illness only based on encounters in 1 year in the EPICON method, we now also used data from previous years to define episodes of illness that started in previous years. Finally, since 2008, it can be determined whether a patient is listed at a general practice based on claims data per quarter of a year, which made the estimation of the size of the studied population (and population at risk) easier and more accurate, resulting in more precise morbidity rates. As a consequence, the new algorithm results in higher prevalence and incidence rates due to a smaller denominator caused by accurately estimated person years and a larger numerator for prevalence rates with the use of disease episodes that started in previous years.

### Validation of the Algorithm

Ideally, a gold standard is needed to test the validity of the duration of the constructed episodes of illness by the algorithm. Besides practical issues (ie, collecting data on a large number of patients for almost 700 diseases), it is for most diseases almost impossible to accurately estimate date of diagnosis and date of recovery. Since the algorithm was developed by experts in the fields of general practice, epidemiology, and medical informatics, we think that the algorithm is a face-valid method to construct episodes of illness. All steps between recording information in EHRs and, eventually, calculating morbidity rates [20] were taken into account during the development of the algorithm. Also, since 2014 the RIVM has accepted our algorithm to estimate national morbidity rates for evaluating health policy [23] and calculating trend scenarios about how many people will have one or more chronic diseases in 2040 [24] for the Dutch Ministry of Health.

An alternative, more indirect, approach to test the validity of the algorithm is to compare our morbidity rates with estimates based on epidemiological studies in the Netherlands. Although the use of different (definitions of) numerators and denominators makes good comparisons difficult [25], estimates in this study are in line with other reported rates of, for instance, diabetes mellitus [26], chronic obstructive pulmonary disease [27], and dementia [28].

Adding half of the duration of the contact-free interval to the date of the last encounter to estimate the date of recovery can result in overestimation or underestimation of the duration of the episode of illness in individual patients. Since the algorithm is used to estimate morbidity rates on group level, we do not expect this approach will affect the results negatively.

### Implementation of the Algorithm in Other Settings

Since the algorithm is developed based on a GP registry, the algorithm and method to calculate morbidity rates will provide the most valid morbidity estimates in GP registries in countries where GPs have a gatekeeper role (eg, Netherlands, United Kingdom, Spain, and Italy) with a fixed patient list, including information from patients who do not visit their GP on a regular basis. In these settings, the GP has the best overview of all health problems in their patient population. In health care systems without a GP (eg, United States), the algorithm is more difficult to implement.

The availability of recorded episodes of care is not essential for using the algorithm. However, compared with data from registries based on single encounters, the start date of an episode of illness will be more precise for chronic diseases (date of diagnosis versus date of first recorded encounter for the disease).

We believe that the algorithm can easily be applied in other registries and settings. In order to construct episodes of illness with the algorithm, apart from a (preferably fixed) patient list, diagnostic data and a corresponding recording date are the only information needed. However, validity of the morbidity rates based on these constructed episodes of illness depends on the population used, data quality, and validity of the recorded diagnostic information, among other things. Also, the algorithm can be used with a combination of various heterogeneous sources. After linking data sources on an individual level, it is essential to develop methods to combine different types of diagnostic information (eg, a combination of ICPC-1 and *International Statistical Classification of Diseases and Related Health Problems, Tenth Revision*, or ICD-10 codes). In this study, we used recorded morbidity data based on ICPC-1 codes. A comparable algorithm can also be developed for other recording methods like ICD codes. In that case, all diseases not included in the ICPC-1 codes need to be categorized in one of the five disease groups with accompanying contact-free intervals.

Finally, in this study a 3-year period was used to estimate morbidity rates. Since not all patients visit their GP for a particular disease on a yearly basis, using a shorter period of time makes it more difficult to distinguish between incident and prevalent cases and could also result in underestimating the number of prevalent cases in the studied population.

### Limitations

The goal of the developed algorithm is optimal use of all recorded data to construct episodes of illness with a more precise estimate of the disease duration. However, this algorithm cannot solve all problems concerning data quality. Also, after selecting the best recording GPs, it still remains unclear whether GPs record all presented morbidity in their EHRs and whether all morbidity is recorded with the correct ICPC code. When patients with more complex diseases are diagnosed and treated in specialized care, it is unclear whether the diagnosis is recorded (with the correct date of diagnosis) in the EHRs of GPs. Because of the gatekeeper role of the GP in the Netherlands, we expect that the diagnostic information from secondary care is also available in EHRs of GPs, since medical specialists keep GPs updated with information about their treatment. Linkage with other registries, especially data from secondary care, could give more insight in the validity of recorded diagnoses by GPs.

In this study, we estimated morbidity rates with data from health care providers. As a consequence, the estimated rates are completely based on patients who are in care for their disease. With EHRs, it is not possible to determine not yet detected cases. To get insight on the total number of patients with a disease, it is better to use specific disease registries. However, such registries are rare and not available for all diseases.

Finally, we used data over the period 2010-2012 for the development of the algorithm. Since the 2013 NIVEL-PCD dataset has not changed, we do not expect updating would change the findings in this study.

### Conclusion

We developed an algorithm to construct episodes of illness based on routinely recorded primary care EHRs. These episodes of illness can be used to estimate morbidity rates. The algorithm constitutes a simple and uniform way of using EHR data and can easily be applied in other registries, thus eliminating one source of variation in outcomes between registries.

## Appendix

Multimedia Appendix 1 Incidence rate and prevalence proportion (per 1000 person years) for each International Classification of Primary Care–1 code.

Multimedia Appendix 2 Categorization of symptoms and diseases in International Classification of Primary Care–1.

## Abbreviations

EHR: electronic health record
GP: general practitioner
ICD-10: International Statistical Classification of Diseases and Related Health Problems, Tenth Revision
ICPC-1: International Classification of Primary Care version 1
NIVEL: Netherlands Institute for Health Services Research
NIVEL-PCD: NIVEL Primary Care Database
RIVM: National Institute for Public Health and the Environment
