# Supplementary material for: Estimating Morbidity Rates Based on Routine Electronic Health Records in Primary Care: Observational Study
Source: JMIR Med Inform. 2019 Jul 26;7(3):e11929. doi: 10.2196/11929 (PMC6688441; doi:10.2196/11929)
Supplement: Multimedia Appendix 1 [file medinform_v7i3e11929_app1.docx]

**Additional file 1: Incidence rate and prevalence proportion (per 1,000 person years) for each ICPC-1 code**

| **ICPC-1** | **Description** | **Incidence rate** | **Prevalence rate** |
| --- | --- | --- | --- |
| A01 | Pain: generalized/unspecified | 4.5 | 5.2 |
| A02 | Chills | 0.3 | 0.3 |
| A03 | Fever | 12.6 | 11.6 |
| A04 | General weakness/tiredness/ill-feeling (excl. pshychol.) | 38.4 | 35.3 |
| A05 | General deterioration (excl. pshychol.) | 1.7 | 2.6 |
| A06 | Fainting (syncope), loss of consciousness | 8.6 | 8.2 |
| A07 | Coma | 0.1 | 0.1 |
| A08 | Swelling (excl. edema K07) | 1.8 | 1.7 |
| A09 | Sweating problems | 2.4 | 3.9 |
| A10 | Bleeding, site NOS | 2.1 | 1.9 |
| A12 | Allergy/allergic reaction NOS | 10.3 | 18.5 |
| A13 | Concern about drug reaction | 5.9 | 5.1 |
| A14 | Infantile colic | 0.2 | 0.2 |
| A15 | Excessive crying infant | 0.9 | 0.9 |
| A16 | Irritable/fidgety infant | 0.1 | 0.1 |
| A17 | Other general sympt. of infants nec | 0.4 | 0.4 |
| A20 | Euthanasia request/discussion | 2.3 | 2.2 |
| A25 | Fear of death/dying | 0.1 | 0.1 |
| A26 | Fear of cancer NOS | 0.7 | 0.7 |
| A27 | Fear of other disease NOS | 1.8 | 1.7 |
| A28 | Disability/impairement NOS | 0.2 | 0.8 |
| A29 | Other general symptom/complaints | 27.3 | 22.2 |
| A70 | Tuberculosis (excl. Respiratory R70) | 0.1 | 0.2 |
| A71 | Measles | 0 | 0 |
| A72 | Chickenpox | 2.5 | 2.6 |
| A73 | Malaria | 0.1 | 0.1 |
| A74 | Rubella | 0 | 0 |
| A75 | Infectious mononucleosis | 1.2 | 1.2 |
| A76 | Other viral diseases with exanthems | 3.9 | 3.8 |
| A77 | Other viral diseases NOS | 5.2 | 4.9 |
| A78 | Other infectious disease NOS | 3 | 2.9 |
| A79 | Carcinomatosis (unknown primary site) | 0.2 | 0.6 |
| A80 | Accident/injury NOS | 13.8 | 13.5 |
| A81 | Multiple trauma/injuries | 0.3 | 0.3 |
| A82 | Late effects of trauma | 0.1 | 0.2 |
| A84 | Poisoning by medical agent | 0.7 | 0.7 |
| A85 | Adverse effect medical agent | 11.2 | 9.9 |
| A86 | Toxic effect other substances | 0.8 | 0.8 |
| A87 | Complication surgery/medical treatment, X-ray | 2.2 | 2.5 |
| A88 | Adverse effect physical factors NEC (excl. H85) | 1.9 | 1.7 |
| A89 | Effects (late) prosthetic device/appliance NEC | 1.6 | 1.7 |
| A90 | Multiple congenital syndromes/congenital anomalies | 0.1 | 1.7 |
| A91 | Investigation with abnormal results NOS | 10.4 | 8.4 |
| A92 | Toxoplasmosis incl. congenital | 0 | 0 |
| A93 | Premature/immature liveborn infant | 0.6 | 1 |
| A94 | All perinatal morbidity | 0.4 | 0.4 |
| A97 | No disease | 81.9 | 66 |
| A99 | Other genralized diseases/multiple syndromes | 26.8 | 56.4 |
| B02 | Enlarged lymph gland(s) | 4.3 | 3.9 |
| B03 | Other sympt. lymphatic glands | 0.7 | 0.6 |
| B04 | Sympt. blood/blood-forming organs | 0.6 | 0.4 |
| B25 | Fear of aids | 0.2 | 0.2 |
| B26 | Fear of cancer of blood/blood-forming organs/lymphatics/spleen | 0.1 | 0.1 |
| B27 | Fear of other blood/lymph disease | 0.3 | 0.4 |
| B28 | Disability/impairement blood/lymphatics | 0 | 0.1 |
| B29 | Other sympt./complt. blood/blood-forming organs/spleen NOS | 1.1 | 0.8 |
| B70 | Acute lymphadenitis | 1 | 1 |
| B71 | Chronic/non-specific lymphadenitis/mesenteric | 0.4 | 0.3 |
| B72 | Hodgkin's disease | 0.2 | 1.9 |
| B73 | Leukaemia | 0.2 | 1.3 |
| B74 | Other malignant neoplasms blood/lymphatics | 0.1 | 0.8 |
| B75 | Benign/unspecified neoplasms blood/lymphatics | 0.1 | 0.1 |
| B76 | Ruptured spleen | 0.1 | 0.1 |
| B77 | Other injuries blood/lymphatics | 0 | 0.1 |
| B78 | Hereditary haemolytic anaemia | 0.2 | 2.4 |
| B79 | Other congenital anomalies blood/lymphatics | 0 | 0.4 |
| B80 | Iron deficiency anaemia | 5.5 | 10.5 |
| B81 | Pernicious/folate deficiency anemia | 2.4 | 6.2 |
| B82 | Anemia other/unspecified | 3.3 | 5.8 |
| B83 | Purpura/coagulation defects/abnormal platelets | 0.7 | 5.2 |
| B84 | Abnormal white cells | 0.2 | 0.3 |
| B85 | Abnormal unexplained blood test | 16.2 | 12 |
| B86 | Other hematological abnormality | 0.6 | 0.5 |
| B87 | Splenomegaly | 0.1 | 0.2 |
| B90 | HIV-infection (incl. aids/ars) | 0.1 | 1.1 |
| B99 | Other disorders of blood/lymph/spleen | 0.4 | 0.7 |
| D01 | Generalized abdominal pain/cramps | 20.8 | 18.6 |
| D02 | Stomach ache/stomach pain | 21.6 | 25.8 |
| D03 | Heartburn | 7.1 | 8.8 |
| D04 | Rectal/anal pain | 1 | 0.8 |
| D05 | Perianal itching | 3.9 | 3.5 |
| D06 | Other localized abdominal pain | 29.7 | 26.6 |
| D08 | Flatulence, gas pain, belching, windy | 1.2 | 1 |
| D09 | Nausea | 7 | 6.3 |
| D10 | Vomiting | 5.2 | 4.8 |
| D11 | Diarrhea | 16.7 | 14.5 |
| D12 | Constipation | 20.7 | 37.9 |
| D13 | Jaundice | 0.3 | 0.3 |
| D14 | Haematemesis/vomiting blood | 0.4 | 0.5 |
| D15 | Melaena/black, tarry stools | 0.6 | 0.7 |
| D16 | Rectal bleeding | 4.8 | 4.4 |
| D17 | Incontinence of bowel | 0.8 | 1.2 |
| D18 | Change faeces/bowel movements | 2.7 | 3.1 |
| D19 | Sympt./Complt. teeth, gums | 4.2 | 3.9 |
| D20 | Sympt./Complt. mouth, tongue, lips | 8.7 | 7.7 |
| D21 | Swallowing problems | 2.5 | 2.2 |
| D22 | Worms/pinworms/other parasites | 2.3 | 2.1 |
| D24 | Abdominal mass NOS | 0.7 | 0.8 |
| D25 | Change in abdominal size/distension | 1.1 | 1.2 |
| D26 | Fear of cancer of digestive system/organ | 0.6 | 0.5 |
| D27 | Fear of other digestive disease | 0.4 | 0.4 |
| D28 | Disability impairment | 0 | 0.3 |
| D29 | Other sympt./complt. digestive system | 1.9 | 2.1 |
| D70 | Infectious diarrhea, dysentery | 2.3 | 2 |
| D71 | Mumps | 0.2 | 0.2 |
| D72 | Infectious hepatitis | 0.8 | 1.4 |
| D73 | Other presumed infections of digestive system | 17.1 | 16 |
| D74 | Malignant neoplasm stomach | 0.1 | 0.7 |
| D75 | Malignant neoplasm colon/rectum | 0.9 | 6.5 |
| D76 | Malignant neoplasm pancreas | 0.2 | 0.4 |
| D77 | Malig. neoplasm digest other/NOS | 0.4 | 1.9 |
| D78 | Benign neoplasms | 1.9 | 2.2 |
| D79 | Foreign body through orifice | 0.6 | 0.6 |
| D80 | Other injuries digestive system | 0.2 | 0.2 |
| D81 | Congenital anomalies digestive system | 0.3 | 2.5 |
| D82 | Disease of teeth/gum | 3.4 | 3.1 |
| D83 | Disease of mouth/tongue/lip | 9 | 8 |
| D84 | Disease of oesophagus | 6.8 | 15.9 |
| D85 | Duodenal ulcer | 0.9 | 1 |
| D86 | Other peptic ulcers | 1.1 | 1.3 |
| D87 | Disorder of stomach function/gastritis | 5.7 | 12.3 |
| D88 | Appendicitis | 1.8 | 1.8 |
| D89 | Inguinal hernia | 3 | 5.1 |
| D90 | Hiatus (diaphragm) hernia | 1.5 | 3.6 |
| D91 | Other abdominal hernia | 1.9 | 3.1 |
| D92 | Diverticular disease intestines | 1.8 | 13.8 |
| D93 | Irritable bowel syndrome | 7.8 | 14.8 |
| D94 | Chronic enteritis/ulcerative colitis | 0.7 | 7.5 |
| D95 | Anal fissure/perianal abscess | 4.2 | 7.2 |
| D96 | Hematomegaly | 0.1 | 0.1 |
| D97 | Cirrhosis/other liver disease | 0.8 | 5.1 |
| D98 | Cholecystitis/cholelithiasis | 3.4 | 6.1 |
| D99 | Other disease digestive system | 2.6 | 4.6 |
| F01 | Eye pain | 1.1 | 1 |
| F02 | Red eye | 3.8 | 3.3 |
| F03 | Discharge from eye | 2.9 | 2.6 |
| F04 | Floaters/spots | 2.6 | 2.4 |
| F05 | Other problems with vision (excl. Blindsness F94) | 10.7 | 9.9 |
| F13 | Abnormal sensation of eye | 14.2 | 11.5 |
| F14 | Abnormal eye movements | 0.5 | 0.4 |
| F15 | Abnormal appearance of eyes | 1.8 | 1.6 |
| F16 | Sympt./complt. of eyelids | 4.8 | 3.9 |
| F17 | Sympt./complt. glasses | 0.3 | 0.3 |
| F18 | Sympt./complt. contact lens | 0.2 | 0.2 |
| F27 | Fear of eye disease | 0.3 | 0.3 |
| F28 | Limited function/disability | 0.2 | 1.4 |
| F29 | Sympt./complt. of eye | 2.3 | 2 |
| F70 | Infectious conjunctivitis (viral/bacterial) | 19.6 | 17.5 |
| F71 | Allergic conjunctivitis | 5.3 | 9 |
| F72 | Blepharitis/stye/chalazion | 11 | 12.3 |
| F73 | Other infections of eye (excl. F85, F86) | 3 | 2.8 |
| F74 | Neoplasm of eye/adnexa | 0.2 | 0.2 |
| F75 | Contusion/abrasions/blackeye | 2.4 | 2.3 |
| F76 | Foreign body in eye | 3.6 | 3.4 |
| F79 | Other injuries eye | 2.8 | 2.4 |
| F80 | Blocked lacrimal duct of infant | 0.3 | 0.3 |
| F81 | Other congenital anomalies eye | 0.2 | 1.6 |
| F82 | Detached retina | 0.8 | 1.3 |
| F83 | Retinopathy | 0.9 | 5.1 |
| F84 | Macular degeneration | 0.8 | 4.6 |
| F85 | Corneal ulcer | 1.2 | 1.1 |
| F86 | Trachoma | 0 | 0 |
| F91 | Refractive errors | 5 | 24 |
| F92 | Cataract | 9.8 | 15.9 |
| F93 | Glaucoma | 1.8 | 11.4 |
| F94 | Blindness | 0.1 | 2.1 |
| F95 | Strabismus | 0.8 | 1.3 |
| F99 | Other diseases eye/adnexa | 6.1 | 10.5 |
| H01 | Ear pain/earache | 9.2 | 8.5 |
| H02 | Hearing complaints (excl. H84, H85, H86) | 7.7 | 7.3 |
| H03 | Ringing/buzzing/tinnitus | 3.3 | 5.4 |
| H04 | Discharge from ear | 3.1 | 2.5 |
| H05 | Blood in/from ear | 0.3 | 0.3 |
| H13 | Plugged feeling ear | 3.7 | 3.3 |
| H15 | Concern with appearance of ears | 0.9 | 0.8 |
| H27 | Fear of ear disease | 0.1 | 0.1 |
| H28 | Disability/impairment ear | 0.1 | 0.8 |
| H29 | Other sympt.complt. of ear | 2.3 | 2.1 |
| H70 | Otitis externa | 22.2 | 19.9 |
| H71 | Acute otitis media/myringitis | 20.9 | 19.8 |
| H72 | Serous otitis media | 10.1 | 9.3 |
| H73 | Eustachian salpingitis | 7 | 6.5 |
| H74 | Chronic otitis media, other infections of ear | 2.1 | 1.8 |
| H75 | Neoplasm of ear | 0.1 | 0.1 |
| H76 | Foreign body in ear | 1 | 1 |
| H77 | Perforation tympanic membrane (excl. H71) | 1.2 | 1.9 |
| H78 | Superficial injury of ear | 0.6 | 0.6 |
| H79 | Other ear injuries | 0.7 | 0.6 |
| H80 | Congenital anomalies of ear | 0.1 | 1.1 |
| H81 | Excessive ear wax | 48.8 | 43.6 |
| H82 | Vertiginous syndrome/labyrinthitis/vestibulitis (excl. N17) | 8.7 | 14.4 |
| H83 | Otosclerosis | 0 | 0.8 |
| H84 | Presbyacusis | 1.8 | 14.8 |
| H85 | Acoustic trauma, noise induced deafness | 0.4 | 3 |
| H86 | Deafness | 2.6 | 16.3 |
| H99 | Other diseases of ear/mastoid | 0.5 | 0.8 |
| K01 | Pain attributed to heart | 5 | 3.8 |
| K02 | Pressure, tightness, heavinesse attributed to heart (excl. R02) | 2.6 | 3.2 |
| K03 | Other pain attributed to circulatory system | 0.2 | 0.3 |
| K04 | Palpitations/awareness of heart | 12.6 | 10 |
| K05 | Other abnormal/irregular heartbeat/pulse | 1.4 | 1.7 |
| K06 | Prominent veins | 0.2 | 0.3 |
| K07 | Swollen ankles/oedema | 10.1 | 11.2 |
| K24 | Fear of heart attack | 0.6 | 0.6 |
| K25 | Fear of hypertension | 3.2 | 2.8 |
| K27 | Fear of other disease circulatory system | 2.5 | 2.2 |
| K28 | Disability/impairment circulatory system | 0.1 | 0.6 |
| K29 | Other sympt./complt. heart/circulatory system | 4.9 | 5.4 |
| K70 | Infectious disease of circulatory system | 0.4 | 0.5 |
| K71 | Acute rheumatic fever/chronic rheumatic heart disease | 0.2 | 0.2 |
| K72 | Neoplasm circulatory system | 0.1 | 0.1 |
| K73 | Congenital anomalies heart/circulatory system | 0.2 | 3.2 |
| K74 | Angina pectoris | 3 | 25.4 |
| K75 | Acute myocardial infarction | 4.1 | 12 |
| K76 | Other and chronic ischaemic heart disease | 1.4 | 11.1 |
| K77 | Heart failure | 2.6 | 13.7 |
| K78 | Atrial fibrillation/flutter | 5.8 | 15.2 |
| K79 | Paroxysmal tachycardia | 1.7 | 3.5 |
| K80 | Ectopic beats | 1.2 | 2.1 |
| K81 | Heart murmur | 1.3 | 2 |
| K82 | Pulmonary heart disease | 0.1 | 0.4 |
| K83 | Heart valve disease NOS, non-rheumatic | 2.8 | 5.8 |
| K84 | Other disease of heart | 2.1 | 4.1 |
| K85 | Elevated blood pressure (excl. K86, K87) | 11.8 | 14.3 |
| K86 | Uncomplicated hypertension | 12.6 | 139.9 |
| K87 | Hypertension with involvement target organs | 2 | 23.2 |
| K88 | Postural hypotension | 1.9 | 3 |
| K89 | Transient cerebral ischaemia | 3 | 7.6 |
| K90 | Stroke/cerebrovascular accident | 2.3 | 17 |
| K91 | Atherosclerosis (excl. K76,K90) | 4.2 | 10.1 |
| K92 | Other arterial obstruction/pheriph. vascular disease | 2.2 | 15.2 |
| K93 | Pulmonary embolism | 1 | 2.2 |
| K94 | Pulmonary embolism | 2.8 | 5.2 |
| K95 | Varicose veins of leg (excl. S97) | 8.4 | 14 |
| K96 | Haemorrhoids | 9.1 | 15.4 |
| K99 | Other disease of circulatory system | 3.1 | 6.3 |
| L01 | Neck symptoms/complaints (excl. N01) | 23.6 | 21.5 |
| L02 | Back symptoms/complaints | 31.1 | 27.5 |
| L03 | Low back symptoms/complaints without radiation (excl. L86) | 40.9 | 36.2 |
| L04 | Chest symptoms/complaints | 29.3 | 26.9 |
| L05 | Flank symptoms/complaints | 3.2 | 3.1 |
| L06 | Axilla symptoms/complaints | 1 | 0.9 |
| L07 | Jaw symptoms/complaints | 4.5 | 3.9 |
| L08 | Shoulder symptoms/complaints | 30 | 26.7 |
| L09 | Arm symptoms/complaints | 6.7 | 6.4 |
| L10 | Elbow symptoms/complaints | 4.3 | 3.8 |
| L11 | Wrist symptoms/complaints | 9.7 | 8.2 |
| L12 | Hand/finger symptoms/complaints | 19.7 | 16.9 |
| L13 | Hip symptoms/complaints | 13 | 11.6 |
| L14 | Leg/thigh symptoms/complaints | 19.1 | 17.4 |
| L15 | Knee symptoms/complaints | 32.6 | 29.7 |
| L16 | Ankle symptoms/complaints | 7.1 | 6.7 |
| L17 | Foot/toe symptoms/complaints | 31.1 | 29.3 |
| L18 | Muscle pain | 14.4 | 12.3 |
| L19 | Other sympt./complt. multriple/unspec. muscles | 6 | 5.3 |
| L20 | Sympt./complt. multiple joints | 6.9 | 7.8 |
| L26 | Fear of cancer of musculoskeletal system | 0 | 0 |
| L27 | Fear of other musculoskeletal disease | 0.5 | 0.4 |
| L28 | Disability/impairment musculoskeletal system | 0.6 | 2.8 |
| L29 | Other sympt./complt. musculoskeletal system | 7 | 6.1 |
| L70 | Infections musculoskeletal system | 0.4 | 0.5 |
| L71 | Neoplasm musculoskeletal | 0.8 | 0.9 |
| L72 | Fracture: radius/ulna | 4.8 | 4.4 |
| L73 | Fracture: tibia/fibula | 2.1 | 3.5 |
| L74 | Fracture: hand/foot bone | 6.1 | 6 |
| L75 | Fracture: femur | 1.5 | 2.5 |
| L76 | Fracture: other | 5 | 8.2 |
| L77 | Sprain/strain of ankle | 11.4 | 10.4 |
| L78 | Sprain/strain of knee | 5.8 | 5.6 |
| L79 | Sprain/strain of other joints | 3.1 | 3 |
| L80 | Dislocations | 2.5 | 2.3 |
| L81 | Other injury musculoskeletal system | 25 | 22.1 |
| L82 | Congenital anomalies | 0.9 | 8.8 |
| L83 | Syndromes related to cervical spine | 3.2 | 5.8 |
| L84 | Osteoarthritis of spine | 1.7 | 12.7 |
| L85 | Acquired deformites of spine/scoliosis/kyphosis | 0.7 | 6.9 |
| L86 | Lumbar disc lesion, bakc pain with radiating pain | 15.1 | 28.3 |
| L87 | Ganglion joint/tendon | 4.6 | 4.4 |
| L88 | Rheumatoid arthritis and allied conditions | 1.3 | 13.3 |
| L89 | Osteoarthritis if hip | 2.3 | 19.8 |
| L90 | Osteoarthritis if knee | 3.7 | 27.9 |
| L91 | Other osteoarthritis and allied conditions | 3.6 | 23.2 |
| L92 | Shoulder syndrome | 12 | 20.8 |
| L93 | Tennis elbow, lateral epicondylitis | 5.6 | 9.1 |
| L94 | Osgood/schlatter, other osteochondroses | 0.6 | 1.1 |
| L95 | Osteoporosis | 3.4 | 22.1 |
| L96 | Acute damage meniscus/ligament of knee | 5.7 | 5.1 |
| L97 | Chronic internal knee derangement | 1.4 | 2.5 |
| L98 | Acquired deformity of limbs | 4.4 | 31.1 |
| L99 | Other disease musculoskeletal system/connective tissue | 31.5 | 52.8 |
| N01 | Headache (excl. N02, N89, R09) | 21 | 18.2 |
| N02 | Tension headache | 8 | 6.8 |
| N03 | Pain face | 0.7 | 0.8 |
| N04 | Restless legs | 1.6 | 3.4 |
| N05 | Tingling fingers/feet/toes | 2.3 | 2.2 |
| N06 | Other sensation disturbances and abn. involuntary movements | 5.3 | 4.4 |
| N07 | Convulsions/seizures | 0.6 | 0.6 |
| N16 | Other disturbance sense/smell/taste | 0.6 | 0.5 |
| N17 | Vertigo/dizziness (excl. H82) | 15.7 | 14 |
| N18 | Paralysis/weakness (excl. A04) | 1.1 | 0.9 |
| N19 | Speech disorder | 2.5 | 4.2 |
| N26 | Fear cancer neurological system | 0 | 0 |
| N27 | Fear of other neurological disease | 0.2 | 0.2 |
| N28 | Disability/impairment neurological system | 0 | 0.3 |
| N29 | Other sympt.complt. of neurological system | 1.5 | 1.2 |
| N70 | Poliomyelitis/ other enterovirus | 0 | 0.5 |
| N71 | Meningitis/encephalitis | 0.2 | 0.4 |
| N72 | Tetanus | 0.2 | 0.2 |
| N73 | Other infection neurological system | 0 | 0.1 |
| N74 | Malignant neoplasm neurological system | 0.1 | 0.6 |
| N75 | Benign neoplasm neurological system | 1 | 1.1 |
| N76 | Unspecified neoplasm neurological system | 0.1 | 0.1 |
| N79 | Concussion | 3.3 | 3 |
| N80 | Other head injury (excl. L76) | 3.3 | 3.3 |
| N81 | Other injuries neurological system | 0.5 | 0.6 |
| N85 | Congenital anomalies neurological system | 0.1 | 1.1 |
| N86 | Multiple sclerosis | 0.1 | 1.7 |
| N87 | Parkinsonism/paralysis agitans | 0.4 | 2.7 |
| N88 | Epilepsy | 0.9 | 10.3 |
| N89 | Migraine | 7.7 | 17.4 |
| N90 | Cluster headache | 0.4 | 0.8 |
| N91 | Facial paralysis/bell's palsy | 0.5 | 0.9 |
| N92 | Trigeminus neuralgia | 0.3 | 0.6 |
| N93 | Carpal tunnel syndrome | 4.2 | 7 |
| N94 | Other peripheral neuritis/neuropathia | 5.6 | 9.8 |
| N99 | Other diseases of neurological system | 1.2 | 2.6 |
| P01 | Feeling anxious/nervous/tense/inadequate | 13.8 | 16.3 |
| P02 | Acute stress/transient situational disturbance | 7.6 | 8.7 |
| P03 | Feeling depressed | 8.6 | 10.5 |
| P04 | Feeling/behaving irritable/angry | 2.3 | 2 |
| P05 | Feeling/behaving old, senile/concern with aging | 1.2 | 0.9 |
| P06 | Disturbances of sleep/insomnia | 22.2 | 26.4 |
| P07 | Inhibition/loss/lack of sexual desire/excitement | 0.6 | 0.5 |
| P08 | Inhibition/loss/lack of sexual fulfilment | 1.7 | 1.3 |
| P09 | Concern with sexual preference | 0.1 | 0.2 |
| P10 | Stammering/stuttering/tics | 0.4 | 0.7 |
| P11 | Eating problems in children | 0.4 | 0.7 |
| P12 | Bedwetting/enuresis (excl. U04) | 1.2 | 2.2 |
| P13 | Encopresis/bowel training problem | 0.1 | 0.2 |
| P15 | Chronic alcohol abuse | 2.5 | 5 |
| P16 | Acute alcohol abuse | 0.6 | 1 |
| P17 | Tobacco abuse | 8.6 | 18.5 |
| P18 | Medicinal abuse | 0.7 | 1.7 |
| P19 | Drug abuse | 1.5 | 2.8 |
| P20 | Disturbances of memory/concentration/orientation | 4.4 | 7.7 |
| P21 | Overactive child, hyperkinetic | 3.9 | 8.4 |
| P22 | Other concern with behavior of child | 4.5 | 7.8 |
| P23 | Other sympt./complt. concerning behavior of adolescent | 0.8 | 1.4 |
| P24 | Specific learning problem | 3.6 | 6.3 |
| P25 | Phase of life problem adult | 0.6 | 1 |
| P27 | Fear of mental disorder | 0.2 | 0.2 |
| P28 | Disability/impairment psychological disorder | 0.1 | 0.7 |
| P29 | Other psychological symptoms and complaints | 6.3 | 7.1 |
| P70 | Dementia (incl. senile, alzheimer) | 1.4 | 6.1 |
| P71 | Other organic psychosis | 1.1 | 1.8 |
| P72 | Schizophrenia | 0.2 | 2.9 |
| P73 | Affective psychosis | 0.8 | 1.8 |
| P74 | Anxiety disorder/anxiety state | 7.1 | 15.8 |
| P75 | Hysterical/hypochrondriacal disorder | 0.6 | 1.2 |
| P76 | Depressive disorder | 10.7 | 28.2 |
| P77 | Suicide attempt | 0.8 | 0.9 |
| P78 | Neuraesthenia/surmenage | 7 | 12.4 |
| P79 | Other neurotic disorder | 1.2 | 2.4 |
| P80 | Personality disorder | 1 | 8.3 |
| P85 | Mental retardation | 0.4 | 3.9 |
| P98 | Other/unspecified psychoses | 1.1 | 2.2 |
| P99 | Other mental/psychological disorder | 3.3 | 6 |
| R01 | Pain: attributed to respiratory system (excl. R09) | 0.4 | 0.4 |
| R02 | Shortness of breath/dyspnoea (excl. K02) | 10.8 | 12.2 |
| R03 | Wheezing | 1.7 | 1.5 |
| R04 | Other breathing problem | 4.6 | 3.9 |
| R05 | Cough | 63.9 | 60 |
| R06 | Nose bleed/epistaxis | 5.7 | 5 |
| R07 | Sneezing/nasal congestion/runny nose | 6.7 | 5.2 |
| R08 | Other symptoms/complaints nose | 6.4 | 5.6 |
| R09 | Sinus symptoms/complaints | 4.1 | 3.7 |
| R21 | Throat symptoms/complaints | 21.9 | 19.5 |
| R22 | Tonsils symptoms/complaints | 1.4 | 1.3 |
| R23 | Voice symptoms/complaints | 3.5 | 3.2 |
| R24 | Haemoptysis | 0.7 | 0.7 |
| R25 | Abnormal sputum/phlegm | 0.6 | 0.6 |
| R26 | Fear of cancer respiratory system | 0.2 | 0.2 |
| R27 | Fear of other respiratory disease | 0.4 | 0.4 |
| R28 | Disability/impairment respiratory system | 0.1 | 0.7 |
| R29 | Other sympt./complt. respiratory system | 1.2 | 1.3 |
| R70 | Tuberculosis (excl. A70) | 0.2 | 0.3 |
| R71 | Whooping cough | 1.4 | 1.5 |
| R72 | Strep throat/scarlet fever | 1.6 | 1.5 |
| R73 | Boil/abscess nose | 0.8 | 0.7 |
| R74 | Upper respiratory infection acute | 81.1 | 68.9 |
| R75 | Sinusitis acute/chronic | 29.4 | 26.2 |
| R76 | Tonsillitis acute | 11.6 | 10.5 |
| R77 | Laryngitis/tracheitis/croup acute | 3 | 2.8 |
| R78 | Acute bronchitis/bronchiolitis | 27.4 | 24.8 |
| R80 | Influenza (excl. R81) | 3.8 | 3.7 |
| R81 | Pneumonia | 13.7 | 16.1 |
| R82 | Pleurisy, all types (excl. R70) | 0.2 | 0.2 |
| R83 | Other infection respiratory system | 3.2 | 3.6 |
| R84 | Malignant neoplasm bronchus/lung | 0.8 | 2.7 |
| R85 | Other malignant neoplasm respiratory system | 0.1 | 0.8 |
| R86 | Benign neoplasm respiratory system | 0.5 | 0.5 |
| R87 | Foreign body nose/larynx/bronch | 0.6 | 0.5 |
| R88 | Other injury respiratory | 0.6 | 0.6 |
| R89 | Congenital anomaly respiratory system | 0 | 0.3 |
| R90 | Hypertrophy/chronic infection tonsils/adenoids | 2.4 | 4.1 |
| R91 | Chronic bronchitis/bronchiectasis | 1 | 7.6 |
| R93 | Pleural effusion NOS | 0.2 | 0.3 |
| R95 | Emphysema/chronic obstructive pulmonary disease | 3 | 29.7 |
| R96 | Asthma | 8.1 | 87.5 |
| R97 | Hayfever/allergic rhinitis | 24.1 | 48 |
| R98 | Hyperventilation | 5.8 | 5.2 |
| R99 | Other disease respiratory system | 2.1 | 3.9 |
| S01 | Pain/tenderness of skin | 1.1 | 1 |
| S02 | Pruritus/skin itching (excl. D05, X16) | 12.7 | 10.8 |
| S03 | Warts | 30.3 | 28.6 |
| S04 | Localized swelling/papules/lump/mass/skin/subcut. Tissue | 8.6 | 7.7 |
| S05 | Generalized multiple swelling/papules/lumps/skin/subcut tissue | 1.3 | 1.1 |
| S06 | Localized redness/erythema/rash of skin | 13.1 | 12 |
| S07 | Generalized/multiple redness/erythema/rash of skin | 2.2 | 2.1 |
| S08 | Other changes in skin color | 1.9 | 1.8 |
| S09 | Infected finger/toe; paronychia | 6.7 | 6.3 |
| S10 | Boil/carbuncle/cellulitis localized | 5.9 | 5.2 |
| S11 | Other localized skin infection | 12.1 | 10.4 |
| S12 | Insect bite/sting | 10 | 9.4 |
| S13 | Animal/human bite | 4.2 | 4 |
| S14 | Burn/scald | 4.5 | 4.5 |
| S15 | Foreign body in skin | 2.3 | 2.2 |
| S16 | Bruise/contusion/crushing with intact skin surface | 9.4 | 8.9 |
| S17 | Abrasion/scratch/blister | 8.3 | 7.7 |
| S18 | Laceration/cut | 20.2 | 19.6 |
| S19 | Other skin injury ans subcutaneous tissue | 3.1 | 3 |
| S20 | Corn/callosity | 4 | 3.9 |
| S21 | Skin texture symptoms/complaints | 10.7 | 8.9 |
| S22 | Nail symptoms/complaints | 2.4 | 2.2 |
| S23 | Hair loss/baldness (incl. alopecia) | 4 | 4.4 |
| S24 | Hair/scalp symptoms/complaints | 2.2 | 2.4 |
| S26 | Fear of cancer of skin | 0.2 | 0.2 |
| S27 | Fear of having other skin disease | 0.3 | 0.3 |
| S28 | Disavility/impairment skin and suncutaneous tissue | 0 | 0.3 |
| S29 | Other sympt./complt. skin/subutaneous tissue | 6.6 | 6 |
| S70 | Herpes zoster | 5.1 | 5.9 |
| S71 | Herpes simplex (excl. F85, X90, Y72) | 3.3 | 2.8 |
| S72 | Scabies/other acariasis | 0.6 | 0.6 |
| S73 | Pediculosis and other skin infestation | 0.6 | 0.5 |
| S74 | Dermatophytosis | 45.7 | 42.2 |
| S75 | Moniliasis/monilia infection/candidiasis (excl. X72, Y75) | 10.3 | 9.7 |
| S76 | Other infectious skin dis.nec/erysipelas | 8.7 | 9.8 |
| S77 | Malignant neoplasm of skin | 3.8 | 25.7 |
| S78 | Lipoma | 4.5 | 5.2 |
| S79 | Other benign neoplasms of skin | 15.9 | 17.7 |
| S80 | Other unspecified neoplasm skin | 0.9 | 1 |
| S81 | Haemangioma/lymphangioma | 1.1 | 5.7 |
| S82 | Naevus/mole | 16.1 | 24.7 |
| S83 | Other congenital skin lesions | 0.4 | 2.4 |
| S84 | Impetigo | 13.7 | 12.5 |
| S85 | Pilonidal cyst/fistula | 0.9 | 0.8 |
| S86 | Seborrhoeic dermatitis /other erythematous dermatoses | 8.4 | 15.4 |
| S87 | Atopic dermatitis/other eczema | 11.7 | 79.8 |
| S88 | Contact dermatitis/allergic eczema | 34.8 | 58.4 |
| S89 | Diaper rash | 2 | 1.8 |
| S90 | Pityriasis rosea | 1.7 | 1.6 |
| S91 | Psoriasis w/wo arthropathy | 2.3 | 21.4 |
| S92 | Pompholyx/other disease sweat glands | 1.5 | 2.6 |
| S93 | Sebaceous cyst | 12.2 | 13.9 |
| S94 | Ingrowing toenail/other disease of nail | 6.3 | 5.3 |
| S95 | Molluscum contagiosa | 2.8 | 4.4 |
| S96 | Acne | 7.2 | 14.4 |
| S97 | Chronic ulcer skin/bedsore | 3.4 | 6.1 |
| S98 | Urticaria | 6.5 | 10.9 |
| S99 | Other disease skin/subcutaneous | 21.5 | 35.1 |
| T01 | Excessive thirst | 0.7 | 0.5 |
| T02 | Excessive appetite | 0.1 | 0.1 |
| T03 | Loss of appetite | 0.5 | 0.5 |
| T04 | Feeding problem of infant/child (excl. P11) | 0.7 | 0.8 |
| T05 | Feeding problem of adult (excl. T06) | 0.4 | 0.4 |
| T06 | Anorexia nervosa w/wo bulemia | 0.4 | 0.9 |
| T07 | Weight gain | 0.8 | 0.9 |
| T08 | Weight loss | 4.4 | 4.9 |
| T10 | Lack of expected physiological development/delay/failure to thrive | 1.4 | 2.4 |
| T11 | Dehydration | 0.6 | 0.6 |
| T15 | Thyroid lump, mass | 1 | 0.8 |
| T26 | Fear of cancer of endocrine system | 0 | 0 |
| T27 | Fear of other endocrine/metabolic disease | 1.2 | 1.2 |
| T28 | Disability/impairment endocrine system | 0 | 0.1 |
| T29 | Other sympt./complt. Endocrine/metabolism/nutrition | 0.9 | 0.7 |
| T70 | Endocrine infection | 0.1 | 0.1 |
| T71 | Malignant neoplasm thyroid | 0 | 0.6 |
| T72 | Benign neoplasm thyroid | 0.2 | 0.3 |
| T73 | Other/unspecified neoplasms endocrine system | 0.5 | 0.5 |
| T78 | Thyroglossal duct/cyst | 0.1 | 0.7 |
| T80 | Other congenital anomalies thyroid | 0.1 | 0.9 |
| T81 | Goiter, thyroid nodule wo thyrotoxicosis | 0.4 | 4.3 |
| T82 | Obesity (BMI>30) | 4.8 | 8.9 |
| T83 | Overweight (BMI<30) | 3.7 | 6.9 |
| T85 | Hyperthyroidism/thyrotoxicosis | 1.7 | 4.2 |
| T86 | Hypothyroidism/myxoedema | 2.7 | 25 |
| T87 | Hypoglycaemia | 0.5 | 0.5 |
| T88 | Renal glucosuria | 0.1 | 0.2 |
| T90 | Diabetes mellitus | 4.8 | 64.1 |
| T91 | Vitamin deficiency/other nutritional disorder | 12.9 | 22.3 |
| T92 | Gout | 2.9 | 21 |
| T93 | Lipid metabolism disorder | 8.5 | 67.8 |
| T99 | Other endocrine/metab/nutrit. Disease | 1.9 | 3.7 |
| U01 | Painful urination | 4.7 | 4.2 |
| U02 | Frequent/urgent urination | 9.2 | 10.4 |
| U04 | Incontinence urine (excl. P12) | 6.5 | 11.7 |
| U05 | Other urination problems | 8.8 | 7.3 |
| U06 | Haematuria/ blood in urine | 3.7 | 3.4 |
| U07 | Other symptoms/complaints of urine | 1.6 | 1.5 |
| U13 | Other sypmtoms/complaints bladder | 0.9 | 1.1 |
| U14 | Symptoms/complaints kidney | 0.9 | 1 |
| U26 | Fear of cancer of urinary system | 0.1 | 0.1 |
| U27 | Fear of other urinary disease | 0.6 | 0.6 |
| U28 | Disability/impairment urinary system | 0.1 | 0.6 |
| U29 | Other symptoms/complaints urinary system | 0.6 | 0.7 |
| U70 | Acute pyelonephritis/pyelitis | 1.7 | 1.6 |
| U71 | Cystitis/other urinary infection | 70.2 | 60 |
| U72 | Urethritis, non-specific (excl. X99, Y99) | 0.6 | 0.6 |
| U75 | Malignant neoplasm of kidney | 0.2 | 1.1 |
| U76 | Malignant neoplasm of bladder | 0.4 | 2.8 |
| U77 | Other malignant neoplasm urinary system | 0 | 0.2 |
| U78 | Benign neoplasm urinary tract | 0.1 | 0.1 |
| U79 | Other unspecified neoplasm urinary tract | 0.2 | 0.2 |
| U80 | Injuries urinary tract | 0.1 | 0.1 |
| U85 | Congenital anomalies urinary tract | 0.1 | 1.6 |
| U88 | Glomerulonephritis/nephrosis | 0.1 | 1.4 |
| U90 | Orthostatic albuminuria/proteinuria | 0 | 0 |
| U95 | Urinary calculus (all types/sites) | 3.3 | 5.6 |
| U98 | Abnormal urine test NOS | 2 | 1.7 |
| U99 | Other disease urinary system | 6.6 | 12.6 |
| W01 | Question of pregnancy (excl. W02) | 1.1 | 1 |
| W02 | Fear of pregnancy | 1.1 | 1 |
| W03 | Antepartum bleeding | 0.3 | 0.2 |
| W05 | Pregnancy vomiting/nausea | 0.8 | 0.8 |
| W10 | Morning after pil/postcoital contraception | 0.4 | 0.4 |
| W11 | Family planning/ oral contraception | 20.4 | 42.7 |
| W12 | Family planning/IUD | 9.8 | 16.5 |
| W13 | Family planning/Sterilization female | 0.6 | 1.1 |
| W14 | Family planning/other female | 4.3 | 9.5 |
| W15 | Infertility/subfertility female | 2 | 3.7 |
| W17 | Heavy post-partum bleeding | 0.1 | 0.1 |
| W18 | Other symptoms/complaints of post-partum period | 0.1 | 0.1 |
| W19 | Symptoms/complaints of lactation | 0.6 | 0.6 |
| W20 | Other symptoms/complaints of breast (during pregnancy) | 0.6 | 0.5 |
| W27 | Fear complications of pregnancy/delivery | 0.1 | 0.1 |
| W28 | Disability/impairment of pregnancy | 0.2 | 0.7 |
| W29 | Other symptoms/complaints of pregnancy/childbearing/family planning | 0.4 | 0.4 |
| W70 | Puerperal infection/sepsis | 0.1 | 0.1 |
| W71 | Other Infectious conditions coexisting with pregnancy/puerperium (excl. W70) | 0 | 0 |
| W72 | Malignant neoplasm coexisting with pregnancy | 0 | 0 |
| W73 | Benign neoplasm coexisting with pregnancy | 0 | 0 |
| W75 | Injury complicating pregnancy | 0 | 0 |
| W76 | Congenital anomalies of mother complicating pregnancy | 0 | 0.1 |
| W77 | Other non-obstetrical conditions/diseases affecting pregnancy/childbirth and puerperium | 0.1 | 0.1 |
| W78 | Pregnancy: confirmed | 9.5 | 16.7 |
| W79 | Unwanted pregnancy | 1.1 | 1.7 |
| W80 | Ectopic pregnancy | 0.2 | 0.3 |
| W81 | Toxaemia of pregnancy/(pre)eclampsia | 0.2 | 0.3 |
| W82 | Abortion spontaneous | 2.1 | 1.9 |
| W83 | Abortion induced | 1 | 1 |
| W84 | Pregnancy high risk | 0.5 | 0.9 |
| W90 | Normal delivery liveborn | 10.5 | 9 |
| W91 | Normal delivery deadborn | 0.1 | 0.1 |
| W92 | Complicate delivery liveborn | 2.3 | 2 |
| W93 | Complicate delivery deadborn | 0.1 | 0.1 |
| W94 | Mastitis puerperalis | 1.1 | 1 |
| W95 | Other disorders of breast in puerperium/disorders of lactation | 0.2 | 0.2 |
| W96 | Other complications of puerperium | 0.1 | 0.1 |
| W99 | Other disorders of pregnancy, delivery and puerperium | 0.2 | 0.5 |
| X01 | Genital pain female | 0.7 | 0.6 |
| X02 | Menstrual pain | 2.1 | 4 |
| X03 | Intermenstrual pain | 0.1 | 0.2 |
| X04 | Painful intercourse female | 0.9 | 1.6 |
| X05 | Menstruation absent/scanty | 1.6 | 2.7 |
| X06 | Menstruation excessive | 3.5 | 6.4 |
| X07 | Menstruation irregular/frequent | 3.3 | 5.8 |
| X08 | Intermenstrual bleeding | 2.1 | 3.6 |
| X09 | Premenstrual symptoms | 0.2 | 0.3 |
| X10 | Postponement of menstruation | 2.9 | 2.5 |
| X11 | Menopausal symptoms/complaints | 4.7 | 9.4 |
| X12 | Postmenopausal bleeding | 1.4 | 2.3 |
| X13 | Postcoital bleeding | 0.9 | 1.5 |
| X14 | Vaginal discharge (excl. X08) | 9.4 | 8.4 |
| X15 | Other vaginal symptoms/complaints | 6.4 | 5.8 |
| X16 | Vulval symptoms/complaint | 3.2 | 2.9 |
| X17 | Pelvis symptoms/complaints | 1.1 | 0.9 |
| X18 | Breast pain female | 3.6 | 3.3 |
| X19 | Breast lump/mass female | 5.2 | 4.6 |
| X20 | Nipple symptoms/complaints female | 1.5 | 1.3 |
| X21 | Other breast symptoms/complaints female | 3.6 | 3.3 |
| X23 | Fear sexually transmitted disease female | 5.3 | 5.1 |
| X24 | Fear of sexual dysfunction female | 0.1 | 0 |
| X25 | Fear of genital cancer female | 0.4 | 0.4 |
| X26 | Fear of breast cancer female | 2 | 1.9 |
| X27 | Fear other genital/breast disease female | 0.2 | 0.2 |
| X28 | Disability/impairment female | 0 | 0 |
| X29 | Other sympt./complt. genital system | 1 | 1 |
| X70 | Syphilis female (excl. A90) | 0 | 0 |
| X71 | Gonorrhoea female | 0.2 | 0.2 |
| X72 | Genital candidiasis female | 15.3 | 13.4 |
| X73 | Genital trichomoniasis female | 0.2 | 0.2 |
| X74 | Pelvic inflammatory disease | 0.5 | 0.5 |
| X75 | Malignant neoplasm cervix | 0.2 | 2.2 |
| X76 | Malignant neoplasm breast female | 1.1 | 11.9 |
| X77 | Other malignant neoplasm genital female | 0.3 | 2.4 |
| X78 | Fibroid/myoma (uterus/cervix) | 2 | 2.2 |
| X79 | Benign neoplasm breast female (excl. X88) | 0.6 | 0.7 |
| X80 | Other benign neoplasm female genital | 0.6 | 0.7 |
| X81 | Other unspecified neoplasm female genital | 0.1 | 0.1 |
| X82 | Injury genital female | 0.1 | 0.1 |
| X83 | Congenital anomalies genital female | 0.1 | 0.5 |
| X84 | Vaginitis/vulvitis NOS | 9 | 8.1 |
| X85 | Cervicitis/other cervical disease NOS | 1.1 | 1 |
| X86 | Abnormal cervix smear | 2.5 | 4.3 |
| X87 | Uterovaginal prolapse | 2.9 | 5.9 |
| X88 | Chronic cystic disease breast | 1.3 | 12.5 |
| X89 | Premenstrual tension syndrome | 0.3 | 0.6 |
| X90 | Genital herpes female | 0.9 | 1 |
| X91 | Condylomata acuminata female | 0.9 | 1 |
| X99 | Other diseases female genital system | 2.4 | 4.1 |
| Y01 | Pain in penis | 0.2 | 0.2 |
| Y02 | Pain in testis/scrotum | 1 | 0.9 |
| Y03 | Discharge from penis/urethra | 0.3 | 0.3 |
| Y04 | Other penis symptoms/complaints | 3.2 | 2.7 |
| Y05 | Symptoms/complaints scrotum and testis | 2.4 | 2.2 |
| Y06 | Symptoms/complaints prostate | 3.8 | 4.7 |
| Y07 | Symptoms/complaints potency (excl. P07, P08) | 2.4 | 4.8 |
| Y08 | Other symptoms/complaints sexual function (excl. P07, P08) | 0.3 | 0.2 |
| Y10 | Infertility/subfertility male | 0.7 | 1.1 |
| Y13 | Sterilization male | 1.9 | 3.2 |
| Y14 | Family planning male other | 0 | 0.1 |
| Y16 | Breast symptoms/complaints male | 0.6 | 0.6 |
| Y24 | Fear of sexual dysfunction male | 0.1 | 0.1 |
| Y25 | Fear sexually transmitted disease male | 4 | 4 |
| Y26 | Fear of genital cancer male | 0.7 | 0.7 |
| Y27 | Fear of other genital disease male | 0.1 | 0.1 |
| Y28 | Disability/impairment male genital system | 0.1 | 0.4 |
| Y29 | Other symptoms/complaints male genital system | 1.4 | 1.3 |
| Y70 | Syphilis male (excl. A90) | 0.1 | 0.1 |
| Y71 | Gonorrhoea male | 0.3 | 0.3 |
| Y72 | Genital herpes male | 0.3 | 0.4 |
| Y73 | Prostatitis/seminal vesiculitis | 1.4 | 1.6 |
| Y74 | Orchitis/epididymitis | 1.8 | 1.7 |
| Y75 | Balanitis | 3.2 | 2.7 |
| Y76 | Condylomata acuminata male | 1.1 | 1.2 |
| Y77 | Malignant neoplasm prostate | 0.6 | 5.4 |
| Y78 | Other malign neoplasm male genital | 0.1 | 1 |
| Y79 | Benign neoplasm male genital | 0.1 | 0.1 |
| Y80 | Injury male genital | 0.5 | 0.4 |
| Y81 | Phimosis/redundant prepuce | 1.7 | 2.8 |
| Y82 | Hypospadias | 0.1 | 0.8 |
| Y83 | Undescended testicle/cryptorchism | 0.5 | 0.7 |
| Y84 | Other congenital anomalies | 0.1 | 0.4 |
| Y85 | Benign prostatic hypertrophy | 5.7 | 7.2 |
| Y86 | Hydrocoele | 0.4 | 0.7 |
| Y99 | Other disease male genital incl. breast | 1.6 | 2.7 |
| Z01 | Poverty/financial problem | 0.7 | 0.7 |
| Z02 | Food/water problem | 0.1 | 0.1 |
| Z03 | Housing/neighbourhood problem | 1 | 1.1 |
| Z04 | Social cultural problem | 0.7 | 0.8 |
| Z05 | Working conditions problem, occupational problem | 3.1 | 3.6 |
| Z06 | Unemployment problem | 0.3 | 0.3 |
| Z07 | Education problem | 0.5 | 0.6 |
| Z08 | Social insurance/welfare problem | 0.3 | 0.3 |
| Z09 | Legal/police problem | 0.1 | 0.1 |
| Z10 | Health care system/access/availability problem | 1.5 | 1.6 |
| Z11 | Problem with being ill | 0.4 | 0.4 |
| Z12 | Relationship problem with partner | 7.5 | 8.3 |
| Z13 | Partner's behaviour problem | 0.8 | 0.8 |
| Z14 | Partner illness problem | 2.9 | 3.2 |
| Z15 | Loss/death of partner | 4.3 | 4.9 |
| Z16 | Relationship problem with child | 2 | 2.2 |
| Z18 | Illness problem with child | 1.4 | 1.6 |
| Z19 | Loss/death of child | 0.9 | 1 |
| Z20 | Relationship problem parent/family | 1.4 | 1.6 |
| Z21 | Behaviour problem parent/family | 1.1 | 1.2 |
| Z22 | Illness problem parent/family | 2.4 | 2.6 |
| Z23 | Loss/death parent/family member | 2.7 | 3 |
| Z24 | Relationship problem with friends | 0.4 | 0.5 |
| Z25 | Problems resulting from assaults/harmful events | 1.6 | 1.9 |
| Z27 | Fear of having a social problem | 0.1 | 0.1 |
| Z28 | Social handicap | 0.4 | 2.5 |
| Z29 | Other social problems | 3.2 | 3.5 |
