# Supplementary material for: Estimating Morbidity Rates Based on Routine Electronic Health Records in Primary Care: Observational Study
Source: JMIR Med Inform. 2019 Jul 26;7(3):e11929. doi: 10.2196/11929 (PMC6688441; doi:10.2196/11929)
Supplement: Multimedia Appendix 2 [file medinform_v7i3e11929_app2.docx]

Categorization of symptoms and diseases of ICPC-1.

| Acute symptoms / diseases  (Short)  Contact-free interval: 4 weeks | A02 A03 A07 A08 A10 A14 A16 A17 A71 A72 A73 A74 A76 A77 A86 A88 A92 A95 A96 A97 B02 B03 B29 B70 B71 D10 D11 D19 D20 D22 D70 D71 D73 D79 D82 D83 F02 F04 F14 F16 F18 F70 F75 F76 F79 H01 H04 H05 H13 H15 H76 H78 H79 H81 L06 L07 L10 L11 L12 L77 L81 N79 R06 R21 R72 R73 R74 R76 R77 R80 R87 S04 S05 S09 S10 S11 S12 S13 S14 S15 S16 S17 S18 S22 S71 S73 S84 S89 S90 S94 T01 T11 U01 U07 U80 W03 W10 W20 W70 W94 W95 W96 X10 X18 X19 X20 X82 Y01 Y02 Y03 Y04 Y05 Y16 Y75 Y80 |
| --- | --- |
| Acute symptoms / diseases  (Moderate)  Contact-free interval: 8 weeks | A04 A06 A13 A15 A20 A25 A26 A27 A29 A75 A78 A80 A84 A85 A91 A94 B04 B25 B26 B27 B76 B85 B86 D01 D04 D05 D06 D08 D09 D13 D16 D21 D26 D27 D80 D88 F01 F03 F05 F13 F15 F17 F27 F29 F73 F80 F85 H02 H27 H29 H70 H71 H72 H73 H74 K01 K04 K24 K25 K27 L01 L02 L03 L04 L05 L08 L09 L13 L14 L15 L16 L17 L18 L19 L26 L27 L29 L72 L74 L78 L79 L80 L87 L96 N01 N02 N05 N06 N07 N16 N17 N18 N26 N27 N29 N80 P04 P05 P07 P08 P27 R01 R03 R04 R05 R07 R08 R09 R22 R23 R24 R25 R26 R27 R75 R78 R88 R98 S01 S02 S03 S06 S07 S08 S19 S20 S21 S26 S27 S29 S72 S74 S75 S85 T02 T03 T15 T26 T27 T29 T87 U05 U06 U26 U27 U70 U71 U72 U98 W01 W02 W05 W17 W18 W19 W27 W29 W71 W75 W82 W83 W90 W91 W92 W93 X01 X14 X15 X16 X17 X21 X23 X24 X25 X26 X27 X70 X71 X72 X73 X74 X84 X85 Y08 Y24 Y25 Y26 Y27 Y29 Y70 Y71 Y74 Z27 |
| Acute symptoms / diseases  (Long)  Contact-free interval: 16 weeks | A01 A81 A87 A89 B75 B77 B84 D02 D03 D14 D15 D18 D24 D25 D29 D78 D85 D86 F72 F74 F86 H75 K02 K03 K05 K07 K29 K70 K71 K72 K85 L20 L70 L71 N03 N72 N75 N76 N81 P01 P02 P03 P06 P09 P29 P77 R02 R29 R71 R81 R82 R83 R86 S23 S24 S70 S76 S78 S79 S80 S93 T04 T05 T07 T08 T70 T72 T73 U02 U13 U14 U29 U78 U79 W73 X29 X78 X79 X80 X81 X90 X91 Y06 Y72 Y73 Y76 Y79 Y85 Z01 Z02 Z03 Z04 Z05 Z06 Z07 Z08 Z09 Z10 Z11 Z12 Z13 Z14 Z15 Z16 Z18 Z19 Z20 Z21 Z22 Z23 Z24 Z25 Z29 |
| Long-lasting reversible diseases  Contact-free interval: 1 year | A05 A09 A12 A70 A82 A93 A99 B80 B81 B82 B87 B99 D12 D17 D72 D84 D87 D89 D90 D91 D93 D95 D96 D98 D99 F71 F82 F92 F95 F99 H03 H77 H82 H99 K06 K75 K78 K79 K80 K81 K83 K84 K88 K89 K93 K94 K95 K96 K99 L73 L75 L76 L83 L86 L92 L93 L94 L97 L99 N04 N19 N71 N73 N89 N90 N91 N92 N93 N94 N99 P10 P11 P12 P13 P15 P16 P17 P18 P19 P20 P21 P22 P23 P24 P25 P71 P73 P74 P75 P76 P78 P79 P98 P99 R70 R90 R93 R97 R99 S82 S86 S88 S92 S95 S96 S97 S98 S99 T06 T10 T82 T83 T85 T88 T91 T99 U04 U90 U95 U99 W11 W12 W13 W14 W15 W77 W78 W79 W80 W81 W84 W99 X02 X03 X04 X05 X06 X07 X08 X09 X11 X12 X13 X86 X87 X89 X99 Y07 Y10 Y13 Y14 Y81 Y83 Y86 Y99 |
| Chronic diseases  No contact-free interval | A28 A79 A90 B28 B72 B73 B74 B78 B79 B83 B90 D28 D74 D75 D76 D77 D81 D92 D94 D97 F28 F81 F83 F84 F91 F93 F94 H28 H80 H83 H84 H85 H86 K28 K73 K74 K76 K77 K82 K86 K87 K90 K91 K92 L28 L82 L84 L85 L88 L89 L90 L91 L95 L98 N28 N70 N74 N85 N86 N87 N88 P28 P70 P72 P80 P85 R28 R84 R85 R89 R91 R95 R96 S28 S77 S81 S83 S87 S91 T28 T71 T78 T80 T81 T86 T90 T92 T93 U28 U75 U76 U77 U85 U88 W28 W72 W76 X28 X75 X76 X77 X83 X88 Y28 Y77 Y78 Y82 Y84 Z28 |
